# The Potential Risk of Electronic Waste Disposal into Aquatic Media: The Case of Personal Computer Motherboards

**DOI:** 10.3390/toxics9070166

**Published:** 2021-07-12

**Authors:** Georgios Kalamaras, Maria Kloukinioti, Maria Antonopoulou, Ioanna Ntaikou, Dimitris Vlastos, Antonios Eleftherianos, Stefanos Dailianis

**Affiliations:** 1 Department of Biology, Faculty of Sciences, University of Patras, GR-26500 Rio-Patra, Greece; up1050529@upnet.gr (G.K.); up1048024@upnet.gr (M.K.); 2 Department of Environmental Engineering, University of Patras, GR-30100 Agrinio, Greece; mantonop@upatras.gr (M.A.); dvlastos@upatras.gr (D.V.); 3 Institute of Chemical Engineering Sciences, Foundation of Research & Technology Hellas (ICEHT/FORTH), 10 Stadiou st., Platani, GR-26504 Patras, Greece; ntaikou@iceht.forth.gr; 4 Akrokeramos Sewerage Laboratory, Athens Water Supply and Sewerage Company (EYDAP SA), GR-18755 Keratsini, Greece; aelef@eydap.gr

**Keywords:** algae, e-waste, genotoxicity, hemocytes, human lymphocytes, leachates, motherboard, mussels, stress indices, trace elements

## Abstract

Considering that electronic wastes (e-wastes) have been recently recognized as a potent environmental and human threat, the present study aimed to assess the potential risk of personal computer motherboards (PCMBs) leaching into aquatic media, following a real-life scenario. Specifically, PCMBs were submerged for 30 days in both distilled water (DW) and artificial seawater (ASW). Afterwards, PCMBs leachates were chemically characterized (i.e., total organic carbon, ions, and trace elements) and finally used (a) for culturing freshwater (*Chlorococcum* sp. and *Scenedesmus rubescens*) and saltwater (*Dunaliella tertiolecta* and *Tisochrysis lutea*) microalgae for 10 days (240 h), (b) as the exposure medium for mussel *Mytilus galloprovincialis* (96 h exposure), and (c) for performing the Cytokinesis Block Micronucleus (CBMN) assay in human lymphocytes cultures. According to the results, PCMBs could mediate both fresh- and marine algae growth rates over time, thus enhancing the cytotoxic, oxidative, and genotoxic effects in the hemocytes of mussels (in terms of lysosomal membrane impairment, lipid peroxidation, and NO content and micronuclei formation, respectively), as well as human lymphocytes (in terms of MN formation and CBPI values, respectively). The current findings clearly revealed that PCMBs leaching into the aquatic media could pose detrimental effects on both aquatic organisms and human cells.

## 1. Introduction

It is well known that human-derived pollutants can threaten environmental and human health, thus undermining the social-economic sustainability and prosperity [1,2]. In this context, great effort is systematically carried out for minimizing the environmental and human risk of main waste streams, like municipal and industrial wastes, as well as leachates from open dumpsites and landfills. However, electronic wastes (e-wastes), which have become one of the largest and ever-increasing waste streams worldwide [3], are of great concern since their rational and environmentally sound management (i.e., recycle, reuse, and appropriate transport) is quite complex and remains problematic [4]. The latter is more evident in some developing countries (mainly in Africa and Asia), where legal loopholes and ineffective environmental policies still exist, and the Basel Convention on the Control of Transboundary Movements of Hazardous Wastes and Their Disposal in third countries (i.e., South Africa and Asia) is violated [4,5].

E-wastes refer to all the electric and electronic equipment, along with their parts, which are discarded with no intention of reusing them [6]. The annual production of e-wastes (i.e., obsolete, or non-functional computer components, such as monitors, motherboards, keyboards, mouse, mobile phones and chargers, compact discs, headphones, television sets, air conditioners, and refrigerators, among others) is estimated at almost 50 million tons (Mts) by 2021, showing increasing trends overtime [4]. Most e-wastes are not recycled and disposed together with conventional wastes [7,8]. In fact, there is evidence that only 20% (almost 8.9 Mt) of the collected e-wastes are properly recycled, with approximately 40 Mts to end up in dumpsites and landfills, where they are either buried or burnt [9,10]. In all cases, their entrance into the environment is considered inevitable through leaching and transport with water, air and soil, thus contaminating both surface and groundwater bodies, as well as coastal basins [8,11]. For instance, several monitoring studies performed near e-waste treatment and workshop sites in developing countries revealed air, soil, and surface waters contamination with e-waste leaching components, mainly heavy metals ([12,13,14,15,16,17], as well as other toxic substances such as dioxins, polyaromatic hydrocarbons (PAHs), polychlorinated biphenyl (PCBs), brominated flame retardants (BFRs), polybrominated diphenyl ethers (PBDEs), and polychlorinated dibenzo-p-dioxin furans (PCDD/Fs) [18].

Among e-wastes, personal computers (PCs) are of great concern, due to their short life and replacement, almost every two-three year [19]. Specifically, PC motherboards (PCMBs) represent the biggest printed circuit boards, including basic operational parts (i.e., capacitors, central processing unit/CPU, battery, external connectors and memory slots among others). PCMBs account for almost 6–9% of the total weight of PCs [20], and possess high concentrations of heavy metals, like Ag, As, Au, Cd, Co, Cr, Cu, Hg, Mn, Ni, Pb, Se, U, and Zn, as well as uranium, bromine, Rare Earth Elements (REEs). and Platinum Group Metals (PGMs, like Ir, Pd, Pt and Rh), whose leaching could be harmful to both aquatic biota and human health [11,21,22,23]. Moreover, it has been recently reported that inappropriate handling or primitive recycling processes of e-wastes could lead to the release of dioxins, polybrominated diphenyl ethers (PBDEs), as well as radioactive isotopes [10,24], thus being responsible for air, soil, and water resources contamination [8].

Despite discrepancies, different e-waste leaching methods like the total threshold limit concentration (TTLC) test, the toxicity characteristic leaching procedure (TCLP), as well as the synthetic precipitation leaching procedure (SPLP), have been performed so far, based on different acid and/or low-pH conditions, e-waste crushing, agitation/extraction processes, and time intervals [25,26,27,28,29]. However, although these methods are considered quite efficient to assess the leachability of toxic substances, like metal ions, more realistic and environmentally relevant testing methods, including both chemical and biological approaches, are highly recommended for assessing the real environmental and human impact of e-waste leaching components, following their direct disposal into water bodies [30].

Considering the latter and in accordance with the Agenda 2030 for Sustainable Development, which pointed out e-waste potent threat to the achievement of the Sustainable Development Goals (SGDs), like the assurance of (a) the good health and well-being, through the reduction of chemicals in water (Goal 3), (b) aquatic ecosystems protection (Goal 14), and (c) the access to clean water (Goal 6) [4], the main goal of the present study was to assess the biological effects of personal computer motherboards (PCMBs) leachates on fresh- and marine species, as well as on human lymphocytes, following a more realistic scenario. In the light of this, PCMBs were submerged for 30 days in both distilled water (DW) and artificial seawater (ASW) and thereafter PCMBs leachates, chemically characterized in a first step (i.e., total organic carbon, ions and trace elements), were used for performing biological tests. Specifically, algal bioassays with freshwater and saltwater algal species (i.e., *Chlorococcum* sp. and *Scenedesmus rubescens* as well as *Dunaliella tertiolecta and Tisochrysis lutea* T. ISO, respectively) were performed to assess the impact of PCMBs leachate on their growth. Moreover, mussels of the species *Mytilus galloprovincialis* were also used for assessing PCMBs leachate mediated cellular and oxidative effects, via the estimation of lysosomal membrane integrity (in terms of Neutral red Retention Time/NRRT assay), superoxide anions (^•^O_2_^−^), and nitric oxide (in terms of nitrite/NO_2_^−^) generation, as well as the extent of lipid peroxidation (in terms of malondialdehyde/MDA) in mussel hemolymph/hemocytes. Furthermore, since the presence of genotoxic compounds into PCMB leachates is of great concern, the formation of micronuclei (MN assay) in mussel hemocytes was estimated and further compared with the cytogenotoxic effects occurred in human lymphocytes (via the application of the Cytokinesis Block Micronucleus/CBMN assay), bearing in mind that the quality of the aquatic sources is undoubtedly related to human health.

## 2. Materials and Methods

### 2.1. Ethics Statement

No permits were required for performing experiments with algae and mussels. The application of the CBMN method using human lymphocytes was approved by the Research Ethics Committee of the University of Patras (Ref. No. 6344/26.6.2020).

### 2.2. PC Motherboards, Chemical and Reagents

Obsolete personal computer motherboards (PCMBs) were used for performing the experimental procedure (see Figure S1). All reagents and solvents used were of the highest analytical grade and purity. The chelating polymer resin SPR-IDA (Suspended Particulate Reagent—Iminodiacetate) as a 10% *w/v* suspension in deionized water was supplied by CETAC. For the preparation of reagents and standards, freshly prepared ultrapure water (18.2 MΩ × cm) was used from Barnstead NANOpure Infinity and eVOQUA systems. Ultrapure hydrochloric acid (HCl, 30%), suprapur® nitric acid (HNO_3_, 65%), and suprapur® ammonium hydroxide (NH_4_OH, 25%) were purchased from Merck. Multi-element stock solution, single-element stock solutions of Hg and P as well as ICP-MS Internal Std Mix (Bi, Ge, In, Li, Lu, Rh, Sc, Tb) were obtained from Agilent.

### 2.3. PCMBs Handling and Experimental Procedure

Batch-like leaching tests were performed using discarded/obsolete PCMBs, with no intention of reusing them. In brief, 2 similar PCMBs were submerged in static tanks (final volume 15L, with one PCMB per tank), containing (a) well-aerated distilled water (DW; pH 7.2 ± 1.4, dissolved oxygen: 7–8 mg L^−1^; salinity <0.05, temperature 17–18 °C), and (b) artificial seawater (ASW: pH 8.2 ± 1, dissolved oxygen: 7–8 mg L^−1^; salinity 35, temperature 17–18 °C). No size reduction or dismantling of the PCMBs components was conducted before and during the test. DW selection as leaching medium was based on its further usage for preparing algal culture medium and conducting CBMN assay, thus avoiding interferences and equivocal results, while the CPBM leaching period was 30 days, according to previous studies that showed the presence of PCMBs leaching chemicals into the aquatic media [21,31]. In parallel, tanks containing PCMB-free DW and/or ASW were used as reference ones. Afterwards, PCMBs were removed, and small volumes/aliquots of each tank (PCMB leachates) were used for chemical characterization.

From each tank, four portions (1000 mL in each case) of either PCMB-free or PCMB-DW leachate (N = 4 in each case) were used (a) for the preparation of the freshwater algal culture (BG-11) and (b) for performing the Cytokinesis Block Micronucleus (CBMN) assay in human lymphocytes cultures. Similarly, relevant portions of PCMB-free and PCMB-ASW were used for (a) the preparation of the saltwater algal culture media, while the rest (almost 10 L) was used for the maintenance/exposure of mussels, respectively (see also Figure S1).

### 2.4. Characterization of Leachates

Total organic carbon (TOC), ions (Cl^−^, NO_2_^−^, NO_3_^−^, PO_4_^3−^, Br^−^) and a battery of trace elements (i.e., Al, P, Cr, Mn, Fe, Ni, Cu, Zn, Se, As, Ag, Cd, Sn, Hg, Tl, Pb) were determined in both DW and ASW leaching media, with or without the presence of PCMBs. Ions were determined by an ICS-1500 ion chromatography by Dionex, using an IonPac AS9-HC analytical column (4 × 250 mm) with Na_2_CO_3_ (9 mmol L^−1^) as eluent and flow rate 1 mL min^−1^. TOC was determined by a TOC-V_CSH_ analyzer by Shimadzu. The TOC value was calculated by subtraction of the inorganic carbon (IC) value from the total carbon (TC) of each sample.

Trace elements were determined by an Agilent 8900 Triple Quadrupole ICP-MS/MS (Agilent Technologies, Tokyo, Japan) with an Agilent SPS4 Autosampler and an Agilent Integrated Sample Introduction System (ISIS). For the determination of metals, the employed method was based on ISO 17294-2 testing protocols [32,33]. The operating parameters are shown in Table S1. Multi-element and single-element stock solutions were diluted to create the calibration curves. The calibration standards and samples were finally prepared in an acid matrix of 2.5% *v/v* HNO_3_ and 0.5% *v/v* HCl. Linear regression was confirmed for all selected elements (R^2^ > 0.999). The limits of quantification of our method, including the preparation and analysis steps, are presented in Table S2.

Before the analysis of the leachates, the chelating polymer resin SPR-IDA (Suspended Particulate Reagent—Iminodiacetate) as a 10% *w/v* suspension in deionized water was used for preconcentration/matrix elimination of ASW. The reagent consisted of 10-micron diameter polymer beads derivatized with the chelating agent iminodiacetate. Then, 45 mL of the ASW sample was transferred to a centrifuge tube and 300 μL of the SPR-IDA (suspended particulate reagent iminodiacetate) in the form of a 10% *w/v* suspension were pipetted directly in the sample. The tube was capped, and the contents were mixed. As the samples were preserved with high-purity nitric at a pH of 1.6, high-purity ammonium hydroxide (NH_4_OH, 25%) was added in two steps (75 μL + 60 μL) to adjust the pH to approximately 8. After each addition of NH_4_OH, the samples were well mixed. The SPR-IDA beads were then allowed to settle for approximately one hour. Thereafter, the samples were placed in a centrifuge and spun at 2000 rpm for 10 min. The supernatants were carefully discarded, avoiding any loss of the resins that adhere to the bottom of the tubes. A solution of ultrapure water adjusted to pH 8 with high purity NH_4_OH was then added to the 45-mL mark of the sample tubes and the contents well mixed. The beads were allowed to settle, centrifuged, and the resulting supernatant liquid carefully discarded. Then, 1.5 mL aliquot of 7% *v/v* high-purity nitric acid was added to the bead residue to extract any bound metal ions [34]. Finally, the extract was diluted to 9 mL with ultrapure water.

### 2.5. Algal Bioassays

Algal strains of the freshwater species *Chlorococcum* sp. (strain SAG 22.83), and *Scenedesmus rubescens* (strain SAG 5.95) (Göttingen, Germany), as well as the saltwater species *Dunaliella tertiolecta* (CCAP19/6B) and *Tisochrysis lutea* (T. ISO strain CCAP 927, formerly listed as *Isochrysis* sp., *Isochrysis galbana*) were used for performing algal bioassays. In all cases, fresh- and salt-water algal strains were maintained and cultured in sterilized flasks, containing filtered DW enriched with BG-11 medium (24 ± 1 °C, pH 8.3 ± 0.3), and filtered ASW enriched with f/2 medium without Si (20 ± 1 °C, pH 8.3 ± 0.3, salinity 35) [35,36], under constant illumination (4300 lx). At the late logarithmic phase, a proper amount of each algal strain (1 × 10^4^ cells mL^−1^) was transferred in conical sterilized flasks (volume 200 mL), containing PCBM-free (control cultures) or PCMB-utilized culture media (BG-11 and f/2 prepared with appropriate portions of filtered PCBM leachate in each case). Algal cell number was daily recorded for a period of 240 h (10 days), using a Neubauer hemocytometer, while the growth (μ) and the inhibition rate (%I) were determined according to well-known guidelines and protocols with some modifications [37]. The results are expressed as the mean ± SD from four measurements in each case.

### 2.6. Mussel Collection and Handling

Mussel species *Mytilus galloprovincialis* (5–6 cm long, almost 1 year old) were collected from a mussel farm, located in a well-monitored and characterized nature reserve area (north side of Korinthiakos Gulf, Galaxidi, Greece). The selection of the current area was based on previous studies that showed negligible levels of inorganic and organic pollution [36,38,39]. Mussel collection was performed in February 2020, thus avoiding any interference of spawning with the obtained stress indices [36,40].

Mussels were transferred and laboratory acclimated for 7 days in static tanks (final volume 200 L per tank) containing aerated, recirculated with UV-sterilized and filtered artificial seawater (ASW, temperature 18 ± 1 °C, salinity 35, pH 8.0 ± 0.7, oxygen saturation > 90%). No mortality was observed throughout the acclimation period. Mussels were maintained without feeding during the acclimation period and then daily fed with *Tisochrysis lutea* (almost 2 × 10^6^ cells mL^−1^).

#### 2.6.1. Mussel Exposure

Before the onset of the experimental procedure, groups of mussels (20 individuals per group) were placed in two static tanks, each containing 10 L of CPBM-free ASW (control group of mussels) and 10L of CPBM- ASW leachate (CPBM-treated group of mussels) and further maintained for 4 days (96 h), without feeding, under the conditions mentioned above. Following 24 h of the onset and after the end (96 h) of the experimental procedure, ten individuals per group of mussels (control and CPBM-treated) were removed and finally used for performing stress indices analysis.

In any case, almost 10 mL of hemolymph were withdrawn from the posterior adductor muscle of 10 individuals (N = 10; 1 mL mussel^−1^; control- and CPBM-treated), using a sterile 1 mL syringe with an 18 G1/2′′ needle, containing equal volume of Alseve buffer [41]. A small portion of hemolymph from each mussel per group was used for the determination of lysosomal membrane stability in mussel hemocytes (using the neutral red retention time/NRRT) assay) and the cytogenetic analysis of DNA integrity (using the micronuclei/MN assay) (N = 10 in any case). The remaining mussel hemolymph was divided into 3 subgroups (each subgroup contained pooled hemolymph from 3 individuals per group of mussels) and further used for determining superoxide radicals, nitric oxide (in terms of nitrites) and MDA content.

#### 2.6.2. Estimation of Lysosomal Membrane Stability and MN Frequency in Mussel Hemocytes (NRRT and MN Assays)

Both assays were performed according to the procedures and criteria proposed by UNEP/RAMOGE [42] with minor modifications. In brief, 40–50 μL of hemolymph from each individual per group of mussels (N = 10) were spread on slides, transferred to a lightproof humidity chamber, and allowed to attach. In the case of the NRRT assay, all slides (cell monolayers) were incubated with 40 μg mL^−1^ of the cationic dye neutral red probe (NR) for 15 min in dark. Afterwards, the slides were examined under a light microscope every 15 min (at least 200 granular hemocytes examined in each slide). The period between the NR probe application and the appearance of the first evidence of dye loss from the lysosomes to the cytosol of at least 50% of the examined cells, represented the NRR time for each mussel. NRRT values (expressed as min) are the mean NRR time value ± SD from the analysis of 10 slides, each corresponding to hemolymph extracted by everyone per group of mussels.

For MN assay, all slides (containing agranular hemocytes) were fixed in methanol: acetic acid (3:1), stained with Giemsa (3% *v/v*), and mounted in Canada balsam, before counting under a light microscope (100× magnification, using immersion oil). At least 1000 hemocytes per slide were examined for the detection of micronucleated cells (MN), thus excluding apoptotic and necrotic ones. The results (in terms of ‰ MN frequencies) are the mean ± SD from the analysis of 10 slides, each corresponding to hemolymph extracted by everyone per group of mussels.

#### 2.6.3. Determination of Superoxide Anions (^•^O_2_^−^) in Mussel Hemocytes

Superoxide anions (^•^O_2_^−^) detection and measurement in mussel hemocytes were spectrophotometrically performed, using nitroblue tetrazolium (NBT) as previously reported by Pipe et al. [43] with some modifications. In brief, almost 1 mL of pooled hemolymph from each mussel subgroup was centrifuged (150× *g*, 10 min, 4 °C) and the collected hemocytes were suspended in 1 mL TBS buffer (0.05 M Tris–chloride buffer, pH 7.6, containing 2% NaCl), containing 1 mg mL^−1^ of NBT. Following incubation for 2 h in the dark, cell suspensions were centrifuged (150× *g*, 10 min, 4 °C), washed twice with 300 μL TBS to remove extracellular NBT, and finally fixed with 300 μL of 70% methanol for 10 min. Thereafter, samples were centrifuged and air-dried for 5 min at room temperature. Afterwards, 1 mL of extraction fluid (2 M KOH in DMSO) was added and after incubation for 30 min, samples were measured spectrophotometrically at 620 nm (spectrophotometer PerkinElmer 551). The results are mean ± SD from 3 different measurements, performed in duplicate (technical replicates, N = 3 × 2), and expressed as OD_620 nm_ per milligram of protein, commonly measured using the Coomassie Brilliant Blue G-250-based assay [44].

#### 2.6.4. Determination of NO in Mussel Hemocytes

The detection and measurement of nitric oxides (NO, in terms of nitrites, NO_2_^−^) in mussel hemocytes were performed by the Griess reaction [45]. Specifically, 500 μL of pooled hemolymph from each mussel subgroup were centrifuged at 150× *g* for 10 min at 4 °C and the supernatant was removed carefully. Thereafter, 500 μL of 1% sulfanilic acid in 5% phosphoric acid were added to each sample, incubated at room temperature for 10 min and 500 μL of 0.1% N-napthyl-ethylene- diamine in 5% phosphoric acid was finally added. After 15 min of incubation, the optical density at 548 nm was measured. The molar concentration of nitrite in each sample was determined from standard curves with known concentrations of sodium nitrite (1–100 μmol L^−1^). The results are mean ± SD from 3 different measurements, performed in duplicate (technical replicates, N = 3 × 2) and expressed as nmol NO per milligram of protein.

#### 2.6.5. Evaluation of MDA Content in Mussel Hemolymph

Lipid peroxidation was measured in the hemocytes of mussels as malondialdehyde (MDA) equivalents, which represent a reliable indicator of oxidative damage/stress [46]. The peroxidation of membrane lipids (in terms of malondialdehyde/MDA equivalents) in the mussels’ hemolymph was measured according to the method described by Vlahogianni and Valavanidis [47]. In brief, 1 mL of pooled hemolymph from each mussel subgroup was centrifuged (150× *g*, 10 min, 4 °C) and the supernatant was diluted in 2 mL of trichloroacetic acid (TCA)–thiobarbituric acid (TBA)–HCl [(15%, *w/v* TCA; 0.375%, *w/v* TBA in HCl 0.25 N), containing 0.02% (*w/v*) of butylated hydroxytoluene (BHT) to prevent further peroxidation process. After heating for 15 min at 90 °C, all samples were cooled at room temperature, centrifuged at 300× *g* for 10 min, and finally measured at 535 nm. A molar extinction coefficient (1.5 × 10^5^ M^−1^ cm^−1^) [48] was used for the determination of the MDA concentration. The results are mean ± SD from 3 different measurements, performed in duplicate (technical replicates, N = 3 × 2) and expressed as nmol MDA per milligram of protein.

### 2.7. Cytokinesis Block Micronucleus (CBMN) Assay in Human Lymphocytes

The cytotoxic and/or genotoxic potential of CPBM leachate was assayed in human lymphocytes, via the Cytokinesis Block Micronucleus (CBMN) assay, using cytochalasin-B. The latter is considered as a reliable tool for assessing the presence of cytogenotoxic substances in aquatic media and highly recommended in case of hardly treated mixtures of hazardous substances or priority aquatic pollutants [49,50,51,52,53].

The CBMN assay was performed according to the standard procedures [54]. Specifically, whole blood samples were collected from healthy and non- smoking male donors (20 and 25 years old), previously declared that they were not exposed to radiation, drug treatment, or any viral infection in the recent past. A small portion of whole blood (0.5 mL) per donor, was added to 6.5 mL Ham’s F-10 medium, containing 1.5 mL fetal bovine serum (FBS) and 0.3 mL phytohaemagglutinin (PHA) for the stimulation of cell division. Appropriate volumes of each CPBM-DW leaching portion were added in culture volume (8.8 mL) 24 h after culture initiation, to obtain final concentrations of 0.2, 1, 5, and 10% *v/v*, which are in accordance with previous studies regarding the cytogenotoxic effects of effluents and leachates [39,53,55]. The verification of the CBMN assay method was performed with the use of the cyto-genotoxic mitomycin C (0.05 μg mL^−1^) (positive control, data not shown). After 44 h of incubation, cytochalasin-B (Cyt-B, final concentration of 6 μg mL^−1^) was added to block cytokinesis of dividing cells. Cultures were incubated at 37 °C in a humidified atmosphere of 5% CO_2_ for 72 h and then cells were harvested and collected by centrifugation. A mild hypotonic treatment with a 3:1 solution of Ham’s medium and milli-Q H_2_O was left for 3 min at room temperature which was followed by 10 min fixation (for at least 3 times) with a fresh 5:1 solution of methanol/acetic acid.

Slides with cells were stained with DAPI and microscopically scored (100× magnification, using immersion oil). The MN formation in almost 4000 binucleated cells was scored in each case (1000 cells per culture in each case) (see also Figure S2). Cytotoxicity was calculated by Cytokinesis Block Proliferation index (CBPI), after counting at least 2000 cells in each case (500 cells per culture in each case), according to Equation (1) [54]:

(1) $$CBPI=\left[\frac{\left(\left(Number\;of\;mononucleate\;cells\right)+\left(2xNumber\;of\;binucleate\;cells\right)+\left(3xNumber\;of\;multinucleate\;cells\right)\right)}{\left(Total\;number\;of\;cells\right)}\right]\;$$

The results are mean ± SD from 4 independent measurements. Each measurement was performed in duplicate (technical replicates).

### 2.8. Statistical Analysis

Statistical analyses were performed using the SPSS 25 (IBM Inc., Armonk, NY, USA, 2019) software package. Data sets were checked for homogeneity of variance (Levene’s test of equality of error variances, SPSS Inc. 16) and the significant differences among variables obtained in control and CPBM-treated bioassays were assessed non-parametrically, using the Mann–Whitney u-test (*p* < 0.05).

## 3. Results

### 3.1. Characterization of Leachates

The chemical analysis of samples showed significant differences in both DW and ASW in the presence or the absence of CPMB (Table 1).

Specifically, CPMB- DW leachates showed significantly increased levels of Cl^−^, Br^−^, and TOC, as well as trace elements, like P, Ni, Cu, Zn, and Sn, compared to those determined in CPMB-free DW, where levels were low and/or undetectable (below LOQ values) in most cases. On the other hand, CPBM-ASW leachates showed the presence of Br^−^, and TOC, as well as Cr, Ni and Sn. In both CPMB leachates, levels of both NO_2_^−^, NO_3_^−^, PO_4_^3−^ and trace elements, such as Fe, Mn, Se, As, Ag, Cd, Hg, Tl, and Pb were lower than LOQ values.

### 3.2. Effects of CPBM Leaching on Freshwater and Saltwater Algal Species

**Figure 1:**
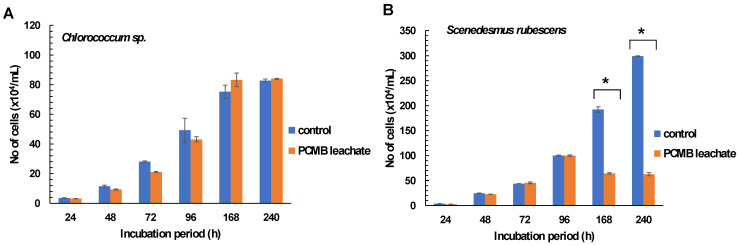
Effects of PCMB-DW leachate in algal species (**A**) *Chlorococcum* sp. and (**B**) *Scenedesmus rubescens*. The results (in terms of number of cells × 10^4^ mL^−1^) are mean ± SDs from 4 independent experiments. Asterisks (*) indicate significant difference between control and e-waste treated algae (Mann–Whitney u-test, *p* < 0.05).

According to the results, freshwater algal species treated with CPBM- DW leachate showed different growth rates over time. Specifically, *Chlorococcum* sp. showed negligible growth rate alterations, with slight attenuation of CPBM leachate inhibitory potential over time (Figure 1A, Table 2A).

On the contrary, in the case of *Scenedesmus rubescens*, although the significant 24 h growth arrest (24 h %I = 21.2± 6.31) declined over time (till 96 h), further treatment showed significantly lower growth rates (high % inhibition rates and low algal densities) than the control (Figure 1B, Table 2B).

**Figure 2:**
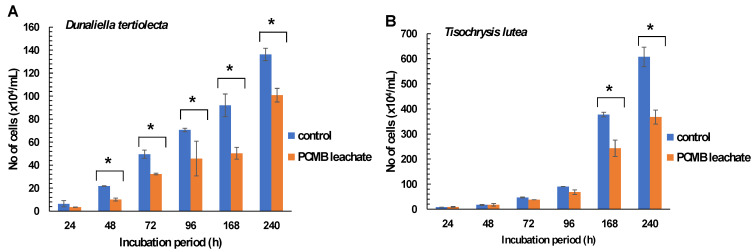
Effects of PCMB-ASW leachate in algal species (**A**) *Dunaliella tertiolecta* and (**B**) *Tisochrysis lutea*. The results (in terms of number of cells × 10^4^ mL^−1^) are mean ± SDs from 4 independent experiments. Asterisks (*) indicate significant difference between control and e-waste treated algae (Mann–Whitney u-test, *p* < 0.05).

Similarly, lower growth rates and algal density were observed in *Dunaliella tertiolecta* treated in CPBM-utilized culture media (48–240 h), while in the case of *Tisochrysis lutea* significantly higher inhibitory rates were observed after treatment for 168 and 240 h (Figure 2A,B, Table 2C,D).

### 3.3. CPBM Leaching Mediated Effects on Mussels

**Figure 3:**
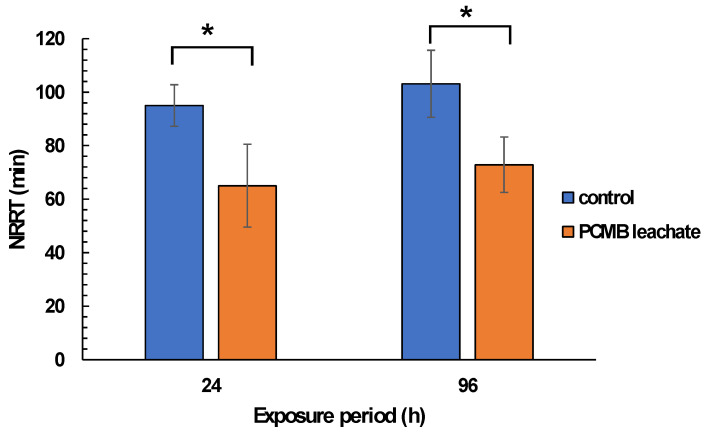
Lysosomal membrane stability (in terms of NRRT values) in hemocytes of mussels exposed to PCMB-ASW for 96 h. The results (expressed as min) are mean± SDs for 10 individuals in each case. Asterisks (*) indicate significant difference from the respective control (Mann–Whitney u-test, *p* < 0.05).

**Figure 4:**
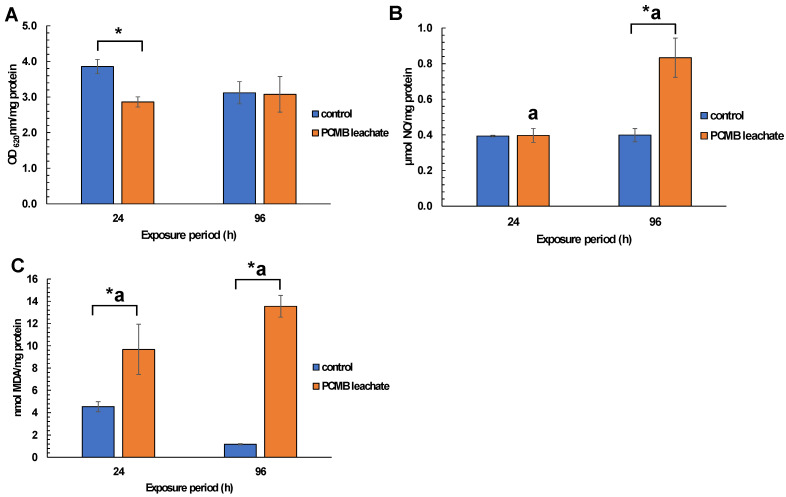
Determination of (**A**) superoxide anions (O_2_^−^), (**B**) nitric oxides (in terms of NO_2_^−^), and (**C**) lipid peroxidation (in terms of MDA equivalents) in hemocytes/hemolymph of mussels exposed to PCMB-ASW for 96 h. The results (expressed as OD_620 nm_ mg protein^−1^, μmol NO mg protein^−1^, and nmol MDA mg protein^−1^, respectively) are mean ± SD from 3 different measurements (each measurement was performed in duplicate) in all cases. Asterisks (*) indicate significant difference from the respective control. Values that share the same letter differ from each other (Mann–Whitney u-test, *p* < 0.05).

**Figure 5:**
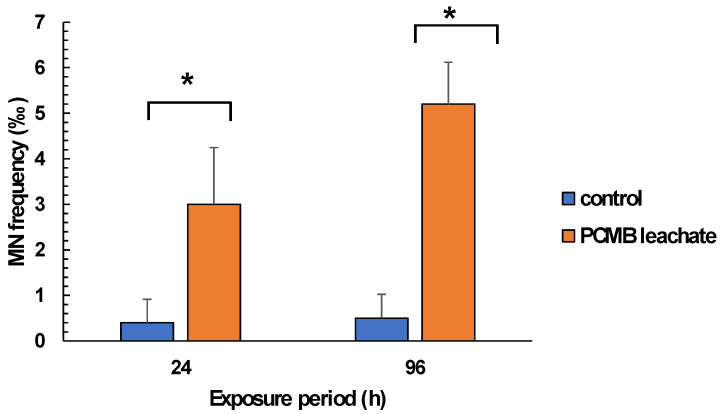
Determination MN frequencies in hemocytes of mussels exposed to PCMB-ASW for 96 h. Values are mean ± SD obtained after analysis of samples derived from 10 individuals of each group of mussels. Asterisks (*) indicate significant difference from the respective control. Values that share the same letter differ from each other (Mann–Whitney u-test, *p* < 0.05).

Regarding mussel exposure to CPBM-ASW leachate, no mortality was observed during the exposure period. However, the results showed significantly lower NRRT values in hemocytes of mussels after the onset (24 h) as well as at the end (96 h) of the exposure period, thus indicating an acute lysosomal membrane destabilization, compared to those that occurred in control mussels (Figure 3). On the other hand, ^•^O_2_^−^ levels remains constant, with a significant increase of NO levels to be observed in hemocytes of 96 h treated mussels (Figure 4A,B). Similarly, an enhanced peroxidation of lipids was observed in the hemolymph of mussels exposed for 24 and 96 h to CPBM leachate (Figure 4C). Interestingly, the latter was characterized by genotoxic potential, as revealed by the high MN frequencies observed in mussel hemocytes (Figure 5).

### 3.4. CPBM Leaching Mediated Effects on Human Lymphocytes (CBMN Assay)

**Figure 6:**
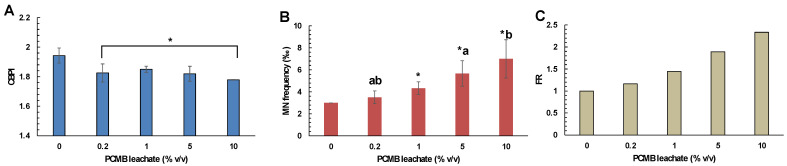
Determination of (**A**) cytotoxic (in terms of CBPI values) and (**B**,**C**) genotoxic (in terms of MN frequency and frequency ratio) effects of PCMB-DW leachate on human lymphocytes. The results are mean ± SD from 4 different measurements in all cases. Asterisks (*) indicate significant difference from the respective control. Values that share the same letter differ from each other (Mann–Whitney u-test, *p* < 0.05).

Considering the CBMN assay, the results showed the CPBM leachate cytotoxic and genotoxic potential against human lymphocytes. Specifically, cells treated with different concentrations of CPBM leachate (0.2–10% *v/v*) showed lower CBPI values, compared to those that occurred in control (Figure 6A). Moreover, cells treated with CPBM leachate at concentrations ranged from 1 to 10% *v/v* showed a significant induction of MN formation, with almost a 2-fold increase to be observed in cells treated with the highest CPBM leachate concentration (Figure 6B,C).

## 4. Discussion

### 4.1. PCMB Chemical Substances in Leaching Media

Based on a real-life scenario, the present study revealed that the direct disposal of PCBMs into fresh- and saltwater bodies could lead to the release of different types of chemical substances, even after a leaching period of 30 days. Similarly, Lithner et al. [31] reported that different discarded electronic products can leach toxic compounds during a short-term (3 days) period of leaching into pure water (pH 7, 23 °C), while Almeida et al. [21] reported that PCMBs trace elements could be leached into DW (pH 4.8) even after the first days of a prolonged leaching period (120 days). According to the latter, it seems reasonable to suggest that even under mild leaching conditions, in terms of constant temperature and natural to alkaline pH values ((pH 7–8, 17 °C), inorganic anions, trace elements, and organic toxicants can be leached by PCMBs into both media tested. The latter was supported by the increased levels of total organic carbon (TOC) measured in both leaching media, as well as the high levels of Cl^−^, Br^−^ and trace elements, such as Al, Cr, Ni, Cu, Zn, and Sn.

Bromide ions (Br^−^) concentrations measured in both PCMB-DW and PCMB-ASW leachates were within the range of values reported by Almeida et al. [21] (1.21 mg/L), following a leaching period of 120 days, with Br^−^ concentrations almost 1 mg/L to be obtained only after 30 days of leaching. The latter supports previous evidence that Br^−^, mainly derived by halogenated organic pollutants (HOPs) like brominated flame retardants (BFRs) typically encountered in MBs, is likely to be one of the major e-waste pollutants in aquatic media [56]. Additionally, the presence of Al, Cr, Ni, Cu, Zn, and Sn as main elements in MBs components (i.e., capacitors, processors, etc.) has been recently reported [18,21,22]. Although different types of organic chemical substances were not measured, the high levels of TOC could depict the presence of organic toxicants, mainly released during the PCMB leaching process. The latter could be detrimental for biota, due to organic carbon’s ability to form complexes with metals and nutrients, thus acting as a substrate for microbial production [57]. Moreover, the increased levels of Cl^−^ mainly derived from chlorinated flame retardants (CFRs) commonly used to produce MB capacitors, could react with organic carbon precursors to form trihalomethane that is harmful to aquatic biota and human [58], but more studies are needed for elucidate the latter.

Differences among the number and the amount of trace elements and inorganic and organic substances leached into DW and ASW is not surprising. In fact, since there is evidence that their leaching depends on both the method and media used, small changes in pH values, metal mobility and chemical form, the presence of organic compounds and corrosion time intervals [25,59,60,61], their presence could be over- or underestimated, depending on the procedure currently used. The latter could lead to the suggestion that the number and the amount of those substances could not be comparable to their presence in solid e-wastes, representing only a small fraction of leaching substances (including both organic and inorganic compounds) that currently occurred after the application of acid-based e-waste leaching methods (i.e., TTLC, TCLP, SPLP methods) [25,62,63]. However, their presence, even under mild leaching conditions currently performed, following corrosion [64], is of great environmental concern, thus giving rise to their detrimental biological effects [21,65].

### 4.2. Biological Effects of PCMB Leachates

Until now, previous studies were focused on the assessment of e-waste ability to promote mortality in tested organisms, as well as challenged species ability to accumulate e-waste leaching substances into their tissues. However, data regarding the assessment of non-lethal mediated effects of e-wastes on species from different trophic levels are scarce. In fact, given that e-waste is considered as a complex mixture of chemical substances, whose inhibitory, synergistic, and additive interactions are questioned and hardly assessed, the investigation of e-waste mediated biological effects requires a battery of biological tests and assays, commonly related with different species as well as aspects of organism function [66]. To this end, the present study showed that PCMB leaching into DW and ASW could affect algal growth, marine mussels, and human health.

#### 4.2.1. Effects on Algal Species Growth and Survival

Algae, representing key producers in the aquatic ecosystems, are ideal biological models for alarming disturbances in water quality due to the presence of different types of chemicals, wastes and leachates ([35,36,37,51,67]. To this end, algae cultured in PCMB leachate-utilized media showed differential responses in their growth and algal cell densities, thus revealing the deterioration of culture media due to chemical substances leaching from PCMB. Considering algal cell densities and growth rates over time, *Chlorococcum* sp. seemed to be less vulnerable towards PCMB leachate, as revealed by the negligible changes of algal cell density and growth rate, while *Scenedesmus rubescens*, bearing different growth rate showed a significant decline of algal cell density over time. Similarly, both saltwater species *Dunaliella tertiolecta* and *Tisochrysis lutea* showed a significant decline in their growth and cell density over time, thus revealing their sensitivity.

Despite the absence of data regarding algal response against such a mixture of contaminants (present in e-waste leachates), differences between algae could be due to cell size, the presence of cell wall, as well as species ability to absorb and eliminate contaminants. For example, algae with cell wall, like *Chlorococcum* sp., could be less vulnerable to chemical substances, thus bearing higher growth rates after prolonged exposure periods, probably due to adaptation and metabolic regulations [51,68]. On the other hand, the presence of hazardous substances at levels almost critical for growth could inhibit or prompt algal growth [69,70], depending on the elimination rates of the tested species [71]. Moreover, there is evidence that chemical substances could disturb cell wall integrity, thus leading to cell division dysfunction [72,73], before algal adaptation occurs over time, as revealed by PCMB-treated *Scenedesmus rubescens* low growth rates and cell density. In case of wall-free *Dunaliella tertiolecta* and *Tisochrysis lutea*, the absence of cell wall could make them sensitive to chemical substances, which in turn could lead to lower growth rate and density almost after a short period of exposure [74,75], as revealed by the results of the present study. However, more studies are needed for verifying the exact mechanisms linked to algal behavior against e-waste leachates, bearing in mind the complex nature of those type of wastes, as well as the presence of different types of chemical substances and interactions among them.

#### 4.2.2. Cellular and Oxidative Effects on Hemolymph/Hemocytes of Mussel Mytilus Galloprovincialis

The determination of stress indices in hemolymph of the species *Mytilus galloprovincialis* is widely used for assessing the type of pressure aquatic ecosystems are faced with, as well as the adverse effects of different types of pollutants (either alone or in mixtures) with great precision and reproducibility [39,41,76,77]. Since mussel hemocytes represent the main immune defensive cells in bivalves [78] the present study showed for the first time that PCMBs leaching into marine media (ASW) could disturb hemocyte lysosomal membrane integrity. The latter is of great interest, considering that lysosomes constitute the main cellular organelles of sequestration and detoxification of xenobiotics, while their functional membrane impairment by different types of single or mixtures of both inorganic and organic chemicals could lead to cellular damage [76,77,79]. In fact, given that abiotic factors (i.e., pH, dissolved oxygen, and salinity) remained constant during the experimental period, lysosomal membrane impairment could be related to the uptake of PCMB leaching toxic substances, which in turn lead to the loss of membrane integrity, the release of lysosomal content into the cytoplasm, and the concomitant cellular death, a process that could affect the overall health status of the organism, as previously mentioned [77,80].

In accordance with the latter, the increased levels of both NO and MDA revealed the enhancement of oxidative-like stress in challenged mussels, possibly due to the presence of pro-oxidant PCBM leaching substances, like metals and lipophilic organic compounds. The latter was further reinforced by previous studies, regarding the oxidative potential of e-waste chemicals and their ability to disrupt immune processes [81,82]. In particular, although both NO and/or •O_2_^−^, formed by aerobic metabolism in mitochondria and microsomes, or the stimulation of immune mechanisms, like the respiratory burst in mussel hemocytes, could be vital for cell protection against oxidant injury, NO overproduction has been linked to many aspects of cellular damage, like peroxidation of membrane lipids, mainly via the formation of hydroxyl radicals (HO) and the toxic intermediate peroxynitrite (ONOO^−^) (see [83] and references therein), a fact that was further revealed by the results of the present study.

#### 4.2.3. Cytogenotoxic Effects on Mussel Hemocytes and Human Lymphocytes

Interestingly, the significant increase of MN frequency in mussel hemocytes as well as the cytotoxic (in terms of low CBPI values) and genotoxic (in terms of high MN frequency values) effects currently observed in human lymphocytes, revealed the presence of cytogenotoxic compounds into the leaching media even under mild leaching conditions currently used. In fact, the enhanced levels of lipid peroxides and NO could merely explain the observed genotoxic effects in mussel hemocytes [83], but the presence of e-waste leaching components could be also responsible for the induction of severe cyto-genotoxicity in challenge organisms. The latter was reinforced by previous studies that reported e-waste leaching components’ ability to oxidize nucleobases, and thus induce DNA methylation, and dysfunction of DNA repair [81,82,84]. Moreover, Grant et al. [85] reported that e-waste leaching substances, such as Polybrominated diphenyl ethers, PAHs, Cr, Ni, and Al, could enhance the formation of nuclear abnormalities in both animals and human beings, a fact that was also confirmed by cytogenetic studies performed in residents of e-waste heavily polluted areas [82,86,87]. Moreover, Uba et al. [88] recently reported that the existence of metal–DNA interactions could disturb the cell cycle and the mitotic process in *Allium cepa*. On the other hand, high levels of organic compounds, as indicated by the high levels of TOC, and Cl^−^ could lead to the formation of THM-derived carcinogenic and mutagenic intermediates [58,89], thus increasing the risk posed by the release of e-wastes into aquatic media. However, considering the difficulties in determining each e-waste leaching component into the receiving media, further studies are important to elucidate the mechanism(s) of e-waste leachate mediated genotoxic effects.

## 5. Conclusions

The present study firstly revealed that PCMBs direct disposal into fresh- and saltwater bodies (mild leaching conditions) could be detrimental not only for aquatic biota but also for human health, via the leaching of both inorganic and organic chemical substances, as well as trace elements of environmental concern. Accordingly, it was mentioned that PCMB leachates could affect both fresh- and saltwater algal biomass and growth rates, a fact that could be linked with disturbances on water quality and the ecological relevance of those key producers of the aquatic ecosystems. The latter was further reinforced by the significant cellular, oxidative, and genotoxic effects firstly observed in challenged mussels of the species *Mytilus galloprovincialis*, as well as human lymphocytes, thus indicating the pressure aquatic ecosystems and human beings are faced with, due to the presence of oxidative and cytogenetic PCMB leaching chemical substances. However, the number and the amount of those PCMB leaching chemical substances could merely represent the true environmental and human impact, since more leaching compounds could be also released, following pH changes due to natural water acidification (i.e., acid rain fallout) and/or the increased trend of e-waste disposal over time. To this end, more studies are needed for elucidating the pH-dependent leachability of e-waste derived trace elements in the presence or absence of organic substances, as well as their potential impact, thus enriching scientific and public awareness in favor of natural sources protection and the concomitant human health.

## Figures and Tables

**Table 1:** Determination of ions, total organic carbon (TOC), and trace elements in DW and ASW in the presence or the absence of CPMB. Results (expressed as mg L^−1^ in case of ions and TOC, and as μg L^−1^ in case of trace elements) are mean ± SD from 3 different measurements. Values that share the same letter differ from each other (Bonferroni, *p* < 0.05).

Ions	DW	PCMB-DW	ASW	PCMB-ASW
Cl^−^	0.119 ± 0.02 ^a^	19.57 ± 0.62 ^a^	33392 ± 758.1	33405 ± 568.2
Br^−^	N.D. ^b^	1.29 ± 0.08 ^b^	N.D. ^c^	1.97 ± 0.27 ^c^
TOC	0.74 ± 0.05 ^d^	17.86 ± 0.81 ^d^	1.13 ± 0.14 ^e^	10.54 ± 0.52 ^e^
**Elements**				
Al	<LOQ	<LOQ	<LOQ	525 ± 109.2
P	<LOQ	8.00 ± 2	<LOQ	<LOQ
Cr	<LOQ	<LOQ	<LOQ	0.23 ± 0.01
Ni	<LOQ	11.9 ± 0.2	<LOQ	10.91 ± 0.56
Cu	<LOQ	1.7 ± 0.1	<LOQ	<LOQ
Zn	<LOQ	668.6 ± 6.1	<LOQ	<LOQ
Sn	<LOQ	7.82 ± 0.13	<LOQ	3.05 ± 0.41

N.D.: Not Detected; <LOQ: below Limit of Quantification.

**Table 2:** Effects of PCMB leachate in freshwater algal species (**A**) *Chlorococcum* sp., (**B**) *Scenedesmus rubescens*, (**C**) *Dunaliella tertiolecta* and (**D**) *Tisochrysis lutea*. The results, in terms of growth rate values (μ) and % inhibition rate values (% I) are mean ± SDs from 4 independent experiments. Values that share the same letter differ from each other (Mann–Whitney u-test, *p* < 0.05).

	Duration Period (h)											
	24		48		72		96		168		240	
	Control	CPBM	Control	CPBM	Control	CPBM	Control	CPBM	Control	CPBM	Control	CPBM
*Chlorococcum* sp.												
μ	1.25 ± 0.07	1.14 ± 0.06	1.22 ± 0.03 ^a^	1.11 ± 0.02 ^a^	1.11 ± 0.06	1.02 ± 0.07	0.97 ± 0.04	0.94 ± 0.01	0.54 ± 0.01	0.55 ± 0.07	0.44 ± 0.01	0.44 ± 0.01
% I	9.03 ± 4.52		8.89 ± 1.57		8.57± 0.59		3.3 ± 1.09		−2.37± 1.28		−0.34± 0.1	
*Scenedesmus rubescens*												
μ	1.32 ± 0.1 ^a^	1.04 ± 0.08 ^a^	1.60 ± 0.01 ^b^	1.56 ± 0.01 ^b^	1.26 ± 0.01	1.27 ± 0.01	1.15 ± 0.03	1.15 ± 0.04	0.66 ± 0.04 ^c^	0.52 ± 0.03 ^c^	0.57 ± 0.01 ^d^	0.41 ± 0.05 ^d^
% I	21.2 ± 6.31		2.31 ± 0.49		−1.02 ± 1.02		0 ± 0.31		20.8 ± 0.47		27.43 ± 0.84	
*Dunaliella tertiolecta*												
μ	1.81 ± 0.43 ^a^	1.23 ± 0.03 ^a^	1.54 ± 0.01 ^b^	1.16 ± 0.06 ^b^	1.3 ±0.02 ^c^	1.16 ± 0.01 ^c^	1.06 ± 0.01 ^d^	0.9 ±0.08 ^d^	0.56 ± 0.01 ^e^	0.49 ± 0.02 ^e^	0.49 ± 0.04	0.46 ± 0.06
% I	32 ± 1.91		24.9 ± 3.98		10.95 ± 0.56		10.88 ± 7.91		13.34 ± 2.28		6.13 ± 1.19	
*Tisochrysis lutea*												
μ	2.08 ± 0.01	2.19 ± 0.21	1.41 ± 0.06	1.42 ± 0.14	1.28 ± 0.01 ^a^	1.21 ± 0.01 ^a^	1.13 ± 0.01 ^b^	0.69 ± 0.02 ^b^	0.74 ± 0.03 ^c^	0.69 ± 0.01 ^c^	0.64 ± 0.06	0.59 ± 0.08
% I	−5.1 ± 10.15		−0.44 ± 10.13		5.53 ± 0.12		7.50 ± 2.26		7.50 ± 2.26		7.85 ± 1.20	

## Data Availability

Data sharing not applicable.

## Supplementary Materials

The following are available online at https://www.mdpi.com/article/10.3390/toxics9070166/s1, Supplementary Figures and Tables are available in SM File (Figures S1 and S2, Tables S1 and S2).
