# Supplementary material for: The Potential Risk of Electronic Waste Disposal into Aquatic Media: The Case of Personal Computer Motherboards
Source: Toxics. 2021 Jul 12;9(7):166. doi: 10.3390/toxics9070166 (PMC8309724; doi:10.3390/toxics9070166)
Supplement: Supplementary file 1 [file toxics-09-00166-s001.zip › toxics-1273671-supplementary.pdf]

toxics

MDPI

Article

Supplementary Materials: The Potential Risk of Electronic Waste Disposal into Aquatic Media: The Case of Personal Computer Motherboards

Georgios Kalamaras, Maria Kloukinioti, Maria Antonopoulou, Ioanna Ntaikou, Dimitris Vlastos, Antonios Eleftherianos and Stefanos Dailianis

Table S1. Agilent 8900 ICP-MS/MS parameters and operating conditions.

Instrument parameter	Operating Condition			
RF applied power (W)	1550			
Sampling depth (mm)	10			
Sampling period (sec)	0.31			
Integration Time (sec)	0.1			
Plasma mode	General Purpose			
Nebulizer gas flow rate (L min $^{-1}$)	1.07			
Auxiliary gas flow rate (L min $^{-1}$)	0.90			
Makeup gas flow rate (L min $^{-1}$)	0.00			
Plasma gas flow rate (L min $^{-1}$)	15.0			
Nebulizer	Micromist glass nebulizer			
Nebulizer pump (rpm)	0.1			
Spray chamber	Scott type-double pass			
Cell gas	No gas	He (mL min $^{-1}$)	O_2	H_2 (mL min $^{-1}$)
Gas flow rate (mL min $^{-1}$)	0	4.3	20%	7.0

Table S2. LOQs of the investigated elements.

Mass	Element	LOQ (μg L $^{-1}$)	Mode	Internal Standard
27	Al	10	No gas mode, Single quadrupole	Sc
31	P	0.2	O_2 mode 20%, mass shift 31--> 47, Triple quadrupole	-
52	Cr	0.2	He mode 4.3 mL min $^{-1}$, Single quadrupole	Sc
55	Mn	0.2	He mode 4.3 mL min $^{-1}$, Single quadrupole	Sc
56	Fe	10	H_2 mode 7ml/min, Triple quadrupole	Ge
60	Ni	0.2	He mode, Single quadrupole	Sc

63	Cu	0.2	He mode, Single quadrupole	Ge
66	Zn	10	He mode, Single quadrupole	Ge
75	As	0.2	O_2 mode 20%, mass shift 75-->91, Triple quadrupole	Rh
78	Se	0.2	H_2 mode 7 mL min^{-1}, Triple quadrupole	Ge
111	Cd	0.2	He mode 4.3 mL min^{-1}, Single quadrupole	Rh
118	Sn	0.2	He mode 4.3 mL min^{-1}, Single quadrupole	Rh
202	Hg	0.2	He mode 4.3 mL min^{-1}, Single quadrupole	Tb
208	Pb	0.2	He mode 4.3 mL min^{-1}, Single quadrupole	Tb

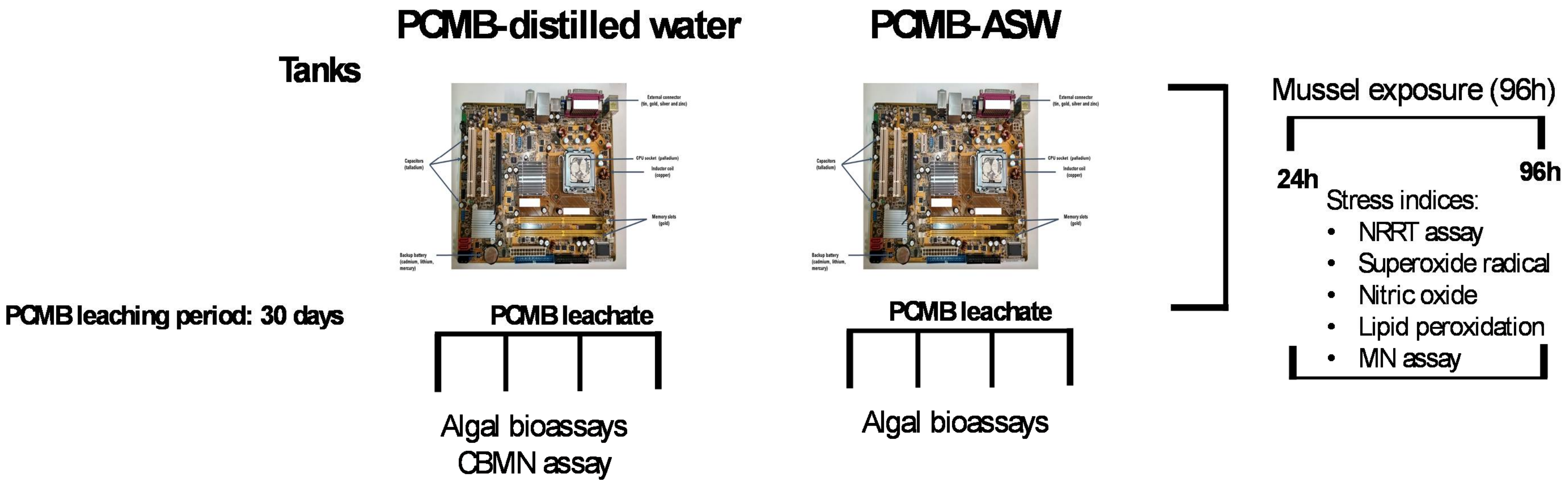

Figure S1. Experimental procedure for assessing the biological effects of PCMB leachates on fresh- and saltwater algal species, mussels, and human lymphocytes (in terms of CBMN assay).

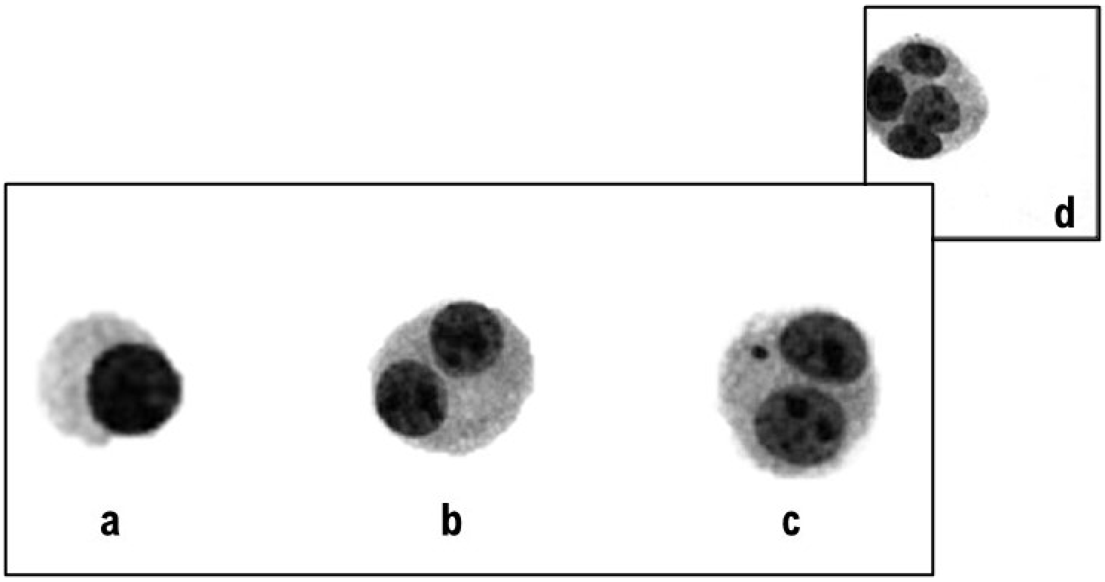

Figure S2. Representative pictures of human lymphocytes; (a) mononucleated cells, (b) binucleated cells, (c) binucleated cells with micronucleus, and (d) polynucleated cells (magnification 1000x).
